# The Flick of a Switch: Conferring Survival Advantage to Breast Cancer Stem Cells Through Metabolic Plasticity

**DOI:** 10.3389/fonc.2019.00753

**Published:** 2019-08-20

**Authors:** Hayley R. Walsh, Brianne M. Cruickshank, Justin M. Brown, Paola Marcato

**Affiliations:** ^1^Department of Pathology, Dalhousie University, Halifax, NS, Canada; ^2^Department of Microbiology and Immunology, Dalhousie University, Halifax, NS, Canada

**Keywords:** breast cancer, metabolic plasticity, CSCs, incRNAs, miRNAs

## Abstract

Within heterogeneous tumors, cancer stem cell (CSC) populations exhibit the greatest tumor initiation potential, promote metastasis, and contribute to therapy resistance. For breast cancer specifically, CSCs are identified by CD44^high^CD24^low^ cell surface marker expression and increased aldehyde dehydrogenase activity. In general, bulk breast tumor cells possess altered energetics characterized by aerobic glycolysis. In contrast, breast CSCs appear to have adaptive metabolic plasticity that allows these tumor-initiating cells to switch between glycolysis and oxidative phosphorylation, depending on factors present in the tumor microenvironment (e.g., hypoxia, reactive oxygen species, availability of glucose). In this article, we review the regulatory molecules that may facilitate the metabolic plasticity of breast CSCs. These regulatory factors include epigenetic chromatin modifiers, non-coding RNAs, transcriptional repressors, transcription factors, energy and stress sensors, and metabolic enzymes. Furthermore, breast cancer cells acquire CSC-like characteristics and altered energetics by undergoing epithelial-mesenchymal transition (EMT). This energy costly process is paired with reprogrammed glucose metabolism by epigenetic modifiers that regulate expression of both EMT and other metabolism-regulating genes. The survival advantage imparted to breast CSCs by metabolic plasticity suggests that targeting the factors that mediate the energetic switch should hinder tumorigenesis and lead to improved patient outcomes.

## Introduction

Relative to non-transformed cells, transformed cells possess unique metabolic requirements (1). Specifically, highly proliferative cells require more energy and biomolecules to support rapid division (1). To accomplish this, transformed cells must increase the production of biomolecules needed and extract elevated quantities of nutrients from the surrounding environment (2). Most cancer cells reprogram metabolic pathways to facilitate the use of aerobic glycolysis as their primary energy source in lieu of oxidative phosphorylation (3). This change is accompanied by an increase in glucose uptake to support the high-energy needs of highly proliferative cancer cells. Although aerobic glycolysis remains inefficient for ATP production, these cells will preferentially initiate aerobic glycolysis even when oxygen is present (Warburg effect) (4). Explanations for this are still unclear; however, some have suggested that the transformed cell switches or reprograms its metabolic machinery to increase biomass instead of maximizing ATP production (5). Others suggest a more integrated approach where cancer cells upregulate glycolysis in response to intermittent hypoxia, producing a large amount of lactate, and increasing the acidity of the microenvironment. In response to this, cancer cells develop acid resistance, which conveys a growth advantage over other cells (3).

For breast tumors specifically, many recent studies are linking glycolytic metabolism to increased tumor progression. For example, plasminogen activator inhibitor 1 (PAI1) promotes migration by stimulating cytoskeletal rearrangement, mitochondrial fragmentation, and glycolytic metabolism in triple-negative breast cancer (TNBC) cells (6). Glycolic metabolite methylglyoxal promotes extracellular matrix remodeling and activation of the pro-migratory signaling pathway, MEK/ERK/SMAD1 in estrogen receptor-positive (ER+) and TNBC cell lines (7). Inversely, treatment of breast cancer cells and xenografts with natural compound cantharidin inhibited migration, invasion, angiogenesis, and metastasis by increasing oxygen consumption and suppressing aerobic glycolysis (8). Furthermore, increased oxygen consumption and dependence on oxidative metabolism through the expression of good prognostic marker B-cell lymphoma two associated death promoter (BAD) resulted in large but non-aggressive breast tumors (9).

Conversely, there is also evidence to support the hypothesis that impeding oxidative phosphorylation with resveratrol is detrimental to breast cancer cells (10). Similar findings regarding the requirement for oxidative phosphorylation for cancer progression have also been reported (11–13). A possible explanation for the contradicting evidence is that cancer cells can express a hybrid phenotype where both glycolytic and oxidative states co-exist, allowing these cells to adapt to the dynamic tumor microenvironment (14). Undoubtedly, distinct subpopulations of cancer cells within tumors (e.g., cancer stem cells, CSCs, vs. non-CSCs), that have a varying capacity for tumorigenicity, metastasis, and proliferation, would also differ in their energetic and metabolic needs. The shifting energy demands inherent of the evolving tumor microenvironment is best met with metabolic plasticity, which increasing evidence suggests, is often associated with CSC populations.

## Breast Cancer Stem CELLS: Inherent Metabolic Plasticity

In a heterogeneous tumor consisting of cancer cells and non-cancer cells (e.g., resident fibroblasts, cancer-associated fibroblasts (CAFs), immune cells), CSCs are the most tumorigenic cancer cell population, illustrated by their capacity to initiate new tumors with high efficiency (15). Tumor cell populations enriched for CSCs can be defined by cancer-specific cell surface markers (e.g., CD44^high^CD24^low^ for breast cancer). Alternatively, increased aldehyde dehydrogenase (ALDH) activity detected by the Aldefluor assay is commonly used to identify CSCs across tumor types. ALDH1A3 and ALDH1A1 are the primary contributors of the Aldefluor^high^ activity associated with breast CSCs (16). Of the breast cancer subtypes, TNBC/basal-like breast cancers have higher percentages of CSCs, which may contribute to their aggressiveness and the worse patient outcomes associated with these breast cancers (17–23). In terms of mitigating the risk of recurrence, the resistance of CSCs to chemotherapies, radiotherapy, and possibly immunotherapies is an increasing concern (24–29). Therapies that target CSCs may reduce the risk of relapse. Signaling pathways [Notch, Wnt, and Hedgehog (30)] and enzymes [e.g., ALDHs (31)] associated with CSCs, are also mediators of tumorigenicity, metastasis, and therapy resistance and are under investigation as potential avenues for therapeutic intervention (32). In recent years targeting CSCs through their metabolic vulnerabilities has been proposed (33).

CSCs can be distinguished from bulk tumor cells based on their dependence on specific metabolic pathways (1). Cancer cells that regulate their glucose metabolism in response to adverse tumor microenvironments are described as metabolically plastic and typically have CSC-associated characteristics such as metastasis and chemo/radio-resistance (34). There is conflicting evidence regarding the dependence of the CSCs of breast (and other tumor types) on glycolysis vs. oxidative phosphorylation. Reports have demonstrated that chemically inhibiting glycolysis reduces populations of breast cancer cells with CSC characteristics (35, 36). Furthermore, during epithelial-mesenchymal transition (EMT), breast cancer cells undergo glycolytic metabolic reprogramming where they acquire CSC-like characteristics and exhibit increased tumorigenicity (37). This EMT/epigenetic and metabolic reprogramming of CSC-like cells are linked by key EMT-initiating factor Snail. A transcriptional repressor, Snail, recruits methyltransferase G9a (increases repressive H3K9me2 and decreases activating H3K9ac histone marks), and DNA methyltransferases (DNMT) to promoters, resulting in hypermethylated silenced genes. These epigenetically silenced genes include epithelial-associated cell adhesion molecule, E-cadherin, and glycolytic antagonist, fructose-1,6-biphosphatase (FBP1), which contribute to EMT development and the switch to glycolytic metabolism in breast cancer cells (37). Inversely, FBP1 expression prevents Snail-mediated EMT (37). Importantly, downregulation of oxidative phosphorylation is intimately linked to the expression of EMT gene signatures (across 20 different cancers, including breast cancer), and metastatic cancers associated with the worse clinical results (38). Although not specifically studied by Gaude and Frezza (38), the well-established connection between EMT and CSCs (39), may indirectly implicate breast CSCs with decreased oxidative phosphorylation. The most commonly downregulated gene in their comprehensive study was mitochondrial respiratory chain enzyme, succinate dehydrogenase complex subunit D (SDHD) (38). In breast tumors downregulation of SDH complex subunit C (SDHC) is associated with EMT, and expression of pro-EMT transcription factors Twist-related protein 1 (TWIST1) and Snail resulted in lower mitochondrial mass and respiration (40). Furthermore, glycolytic phosphoglucose isomerase (PGI)/autocrine motility factor (AMF) is associated with EMT in breast cancer cells and the expression of mesenchymal markers zinc finger E-box-binding homeobox 1 (ZEB1) and ZEB2 (41). Hence, there are plenty of studies suggesting that there is an intimate link between the shift toward glycolytic metabolism and EMT/CSC-like characteristics.

**Figure 1:**
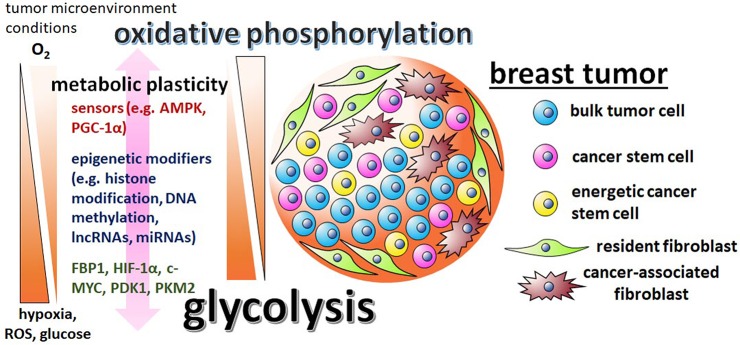
The metabolic plasticity of breast CSCs is dictated by the varying conditions of the tumor microenvironment and mediated by sensors, epigenetic modifiers, and metabolic enzymes. There are contradictory reports regarding breast CSC metabolism, with some studies suggesting the cells are predominately glycolytic, while other studies suggest that breast CSCs utilize primarily oxidative phosphorylation pathways. These discrepancies are possibly reflective of the differing tumor microenvironments in which the cells were assayed in and the metabolic/epigenetic plasticity inherent of CSCs. Influential factors in the tumor microenvironment include oxygen, ROS, and glucose levels. The initiation of the varying metabolic phenotypes associated with breast CSCs depend on regulatory factors, like stress sensors AMPK and PGC-1α, epigenetic modifiers G9a, DNMTs, lncRNA H19, miRNAs let-7 and miR-21, master regulator HIF-1α, glycolytic enzymes PDK1 and PKM2, and glycolytic antagonist FBP1.

In contrast to the studies described above, there is also ample evidence of breast CSCs utilizing oxidative phosphorylation as their primary metabolic program. Vlashi et al. reported that breast CSCs rely on oxidative phosphorylation, while their more differentiated progeny exhibit a glycolytic phenotype (42). Similarly, Banerjee et al. demonstrated that bulk tumor cells depend chiefly on glycolysis, while tumors enriched in breast CSCs rely mainly on oxidative phosphorylation (43). They restricted glycolysis in the breast cancer cells by adapting the cells to fructose, which increased CSCs (i.e., cells with high ALDH activity and the CD44^high^/CD24^low^ phenotype), invasiveness, and chemoresistance. Similarly, a different study showed that breast CSCs are dependent on oxidative phosphorylation (44), while another study suggests breast CSCs rely on alternative metabolic processes such as fatty acid oxidation (45). The contradicting findings concerning breast CSCs displaying predominant glycolytic vs. oxidative metabolism may be a result of the differing microenvironments that the studies were completed in, which in turn evokes the metabolic plasticity phenotype of CSCs (Figure 1). In agreement with this, breast CSC populations demonstrated increased metabolic plasticity by having enhanced ability to boost oxidative phosphorylation upon glycolysis inhibition (46). In the initial culturing of patient-derived lines, CSCs seem to preferentially utilize oxidative phosphorylation rather than aerobic glycolysis (47). Hence it is possible that CSC metabolic reprogramming in a culturing environment that is glucose-enriched, favors glycolysis, while glucose depleted conditions shift the metabolic balance toward oxidative phosphorylation. Determining the cellular factors responsible for metabolic plasticity will allow for more effective targeting of CSC populations within heterogeneous breast tumors.

## Regulators of the Metabolic Phenotypes Associated With Breast Cancer Stem Cells

Essential to metabolic plasticity is the regulation of mitochondria biogenesis. Mitochondria are the hubs that harbor the enzymes that drive the tricarboxylic acid cycle, oxidative phosphorylation, fatty acid oxidation, biosynthesis of nucleotides, amino acids and lipids, and calcium homeostasis (48). Mitochondrial biogenesis is mediated by a diverse signaling network. Key factors include AMP-activated protein kinase (AMPK) and peroxisome proliferator-activated receptor-gamma coactivator 1alpha (PGC-1α), which both function as energy and stress sensors (49). PGC-1α is a co-transcriptional regulator that induces mitochondrial biogenesis by activating transcription factors such as peroxisome proliferator-activated alpha (PPARα), estrogen-related receptor alpha (ERRα), nuclear respiratory factor 1 (NRF1), and NRF2 in breast cancer (50). Increased PGC-1α leads to increased mitochondrial biogenesis, oxidative phosphorylation, and generation of ATP. The requirement of PGC-1α for the oxidative phosphorylation energetics associated with CSCs was first shown in pancreatic cancer (51). More recently, increased PGC-1α was demonstrated in the proteomics analysis of a newly characterized breast CSC sub-population termed energetic cancer stem cells [e-CSCs, Figure 1, Table 1; (59)]. These breast CSCs are characterized by increased glycolysis, oxidative phosphorylation, and mitochondria size.

**Table 1:** Factors that promote or regulate the metabolic reprograming of CSCs or cancer cells with stemness features in breast tumors.

**Factor/regulatory molecule(s)**	**Induced metabolic process(es)**	**Mechanism of action**
AMPK	Glycolysis, oxidative phosphorylation	Master metabolic regulator sensor for adaptations relative to energy and stress levels (46, 49, 52).
EMT gene signature and genes (e.g., Snail, TWIST1, ZEB1, ZEB2)	Glycolysis	Associated with downregulation of oxidative phosphorylation (37–41).
FBP1	Oxidative phosphorylation	Glycolytic antagonist, negative regulator of HIF1α and PKM2 (53). Epigenetic silencing promotes glycolysis, and decreased oxygen consumption and ROS production (37).
FGF13-AS1	Oxidative phosphorylation	LncRNA, inhibits stemness and glycolysis by reducing the half-life of c-Myc transcript (pro-glycolytic and stemness transcription factor) (54).
H19	Glycolysis	In hypoxia, H19 sponges miRNA let-7 leading to upregulation of pro-glycolytic HIF-1α and PDK1 (55).
HIF-1α	Glycolysis, oxidative phosphorylation	Master metabolic regulator and transcription factor that upregulates glucose transporters and glycolytic enzymes, including PDK1 and PKM2 (37, 53, 55, 56). Also promoted the shift toward oxidative epithelial-like CSC state (46).
Hypoxia	Glycolysis, oxidative phosphorylation	Stressor, inducer of pro-glycolytic transcription factors and ncRNAs (37, 55–57). Also promoted the transition to an oxidative epithelial-like breast CSC state (46)
let-7	Oxidative phosphorylation	MiRNA that downregulates pro-glycolytic HIF-1α (55).
miR-21	Glycolysis	MiRNA that regulates both EMT and overexpression of HIF-1α (58).
PDK1	Glycolysis	Glycolytic enzyme, inhibits pyruvate to acetyl CoA conversion, leading to reduced oxidative phosphorylation (55).
PGC-1α	Oxidative phosphorylation	Co-transcriptional regulator that induces mitochondrial biogenesis by activating transcription factors (49–51, 59).
PKM2	Glycolysis	Glycolytic enzyme, production of glycolytic intermediates for biomass production (37, 52).
ROS	Oxidative phosphorylation	Stressor, promotes the transition from a glycolytic mesenchymal-like CSC state to an oxidative epithelial-like breast CSC state (46).
Snail-G9a-DNMT	Glycolysis	Chromatin modifying complex which mediates the repressive histone methylation/deacetylation and DNA methylation, of the FBP1 promoter, which silences the glycolytic antagonist (37).

The metabolic/oxidative cellular stressors hypoxia and reactive oxygen species (ROS) present in the tumor microenvironment drive the need for metabolic plasticity (Table 1, Figure 1). These stressors can promote the transition of ROS^low^ mesenchymal-like quiescent breast CSC state to a ROS^high^ epithelial-like proliferative breast CSC state. Inhibition of glycolysis by 2-deoxy-D-glucose shifted the proportion of CSC populations toward the more oxidative epithelial-like state and decreased the glycolytic mesenchymal-like state (46). This metabolic transition is mediated by activation of the master metabolism regulator axis, AMPK-hypoxia-inducible factor 1 alpha (S-1α) and reversed by the addition of ROS inhibitor N-acetylcysteine (Table 1).

The hypoxia-induced transcription factor HIF-1α is linked to the Warburg effect through its upregulation of glucose transporters, elevated expression of enzymes involved in glycolysis, and inhibition of oxidative phosphorylation, resulting in HIF-1α-mediated glycolysis in cancer cells (60). In breast cancer, negative regulation of HIF-1α by FBP1 results in decreased growth, migration, glucose consumption, and lactate production (53). Epigenetic silencing of FBP1 promoted glycolysis, increased glucose uptake, macromolecule biosynthesis, sustained ATP production under hypoxic conditions, and inhibited oxygen use and ROS production by suppressing mitochondrial complex I activity (Table 1) (37). Importantly, the loss of FBP1 also promoted the CSC-like phenotype in the breast cancer cells by increasing the interaction of Wnt signaling molecules β-catenin and transcription factor t-cell factor (TCF). HIF-1α promotes glycolytic reprogramming at least in part through its regulation of key glycolytic enzymes in glucose metabolism, pyruvate dehydrogenase kinase 1 (PDK1), and pyruvate kinase isoform M2 (PKM2) (56). For example, when PKM2 activity is inhibited by FBP1, glycolytic intermediates for biomass synthesis are not produced (37). Treatment of breast tumor xenografts with diallyl disulfide suppressed breast CSCs and glucose metabolism in a mechanism implicating PKM2 and AMPK (52). In the hypoxic regions of breast tumor xenografts, PDK1 is increased in breast CSC populations, where it activates glycolysis and promotes CSC characteristic in breast tumor xenografts (55). The study on PDK1 also highlighted the essential role that epigenetic modifiers, non-coding RNAs, play in the metabolic plasticity of breast CSCs.

## Non-Coding RNAs As Mediators of Breast Cancer Stem Metabolic Plasticity

Non-coding RNAs are key mediators of metabolic reprogramming, with early evidence illustrating the essential role of microRNAs (miRNAs). For example, hypoxia-induced miR-210 reduces mitochondrial respiration in cancer cells, including breast cancer (57). Similarly, inflammation-induced miR-155 promotes glycolysis by regulating expression of glycolytic enzyme hexokinase two in breast cancer cells (61).

The dysregulation of non-coding RNAs, miRNAs and long non-coding RNAs (lncRNAs), result in gene expression changes that are critical in not only metabolic reprogramming of breast cancer, but also EMT and progression to metastasis, CSC maintenance, and response to therapy (58, 62–64). LncRNAs are non-protein coding transcripts >200 nucleotides in length. Notably, the number of lncRNAs expressed in humans is at least equivalent to the number of protein-coding genes (65, 66). It was once thought that these molecules had little functional relevance; however, characterization of some lncRNAs has revealed they typically function in gene regulation by acting as activators or decoys for transcription factors, guides, recruiters of chromatin-modifying complexes, and miRNA sponges (67). Due to their tissue-specific expression patterns and selective expression in certain cancers, lncRNAs represent attractive therapeutic targets (68).

In terms of evidence for the role of lncRNAs in the metabolic reprogramming of breast cancer, there are several recent examples. LincRNA-p21 plays a critical role in hypoxia-induced glycolysis in breast cancer. The hypoxia-responsive lncRNA binds HIF-1α, preventing its ubiquitination and degradation, leading to the accumulation of the key hypoxic mediator of glycolysis (69). Similarly, lactate-induced HIF-1α-stabilizing long non-coding RNA (HISLA) blocks the interaction of prolyl hydroxylase domain-containing protein 2 (PHD2) and HIF-1α, which inhibits the hydroxylation and degradation of HIF-1α (70). In TNBC, long intergenic non-coding RNA for kinase activation (LINK-A) promotes glycolysis by mediating phosphorylation and stabilization of HIF-1α (71).

For breast CSCs specifically, the evidence that miRNAs and lncRNAs play essential roles in mediating the metabolic plasticity of the cells is starting to accumulate. This is well-exemplified by how they mediate the effects of PDK1 and HIF-1α in breast CSC-specific glycolysis (55). LncRNA H19 sponges miRNA let-7 and HIF-1α is a target of the miRNA. The sponging of let-7 by H19 resulted in upregulation of HIF-1α, which then increased the expression of glycolytic enzyme PDK1. Therefore, the dysregulation of the lncRNA H19 is the initiating event that results in that metabolic reprogramming of CSCs under hypoxic conditions. Similarly, HIF-1α is regulated by miR-21 in the CSC-like cells of breast cancer cells, with antagonism of the miRNA suppressing EMT, CSC-like qualities and HIF-1α (58). FGF13-AS1 may play a critical role in the glycolytic reprogramming of breast CSCs. This lncRNA is downregulated in breast tumors and inhibits glycolysis and stemness by reducing the half-life of c-Myc mRNA (54). Stemness-associated c-Myc is a transcription factor and target of HIF-1α and induces the expression of glycolytic genes, leading to altered glucose consumption (72). It is very likely that as the functions of more lncRNAs are revealed, further connections between these epigenetic regulatory molecules, stemness, and altered energetics in breast cancer will continue to be made.

## Conclusions

Most evidence supports the hypothesis that within a heterogeneous tumor, breast CSCs likely occupy a unique metabolic state relative to other cancer cells, mediated by the increased epigenetic and metabolic plasticity of the cells. Like other solid tumors, breast cancers generally possess genetic modifications that facilitate increased glycolysis in the presence of ample glucose, and under normoxic conditions, CSCs can utilize oxidative phosphorylation. Hence, due to the metabolic adaptability of CSCs, targeting a single metabolic pathway is likely insufficient for tumor eradication. For example, glycolytic inhibitors such as dichloroacetate and phloretin are proposed novel anti-cancer drugs that rely upon the metabolic reprogramming of cancer cells (73–75). However, the results of a recent phase I/II clinical trial reported modest responses when patients with tumors with altered metabolic activity were treated with targeted pentose phosphate pathway, glycolysis, and amino acid depleting inhibitors (76). It is unclear how breast CSC populations would be affected by such treatments; however, based on current knowledge of the metabolic plasticity of the cells, it seems unlikely that CSCs would be depleted by such strategies. This suggests that targeting breast CSCs based on the metabolic reprogramming of tumors requires identification of the factors that impart CSCs with metabolic plasticity (Table 1). For example, to target key metabolic plasticity reprogrammer HIF-1α, various drugs have been proposed; aminoflavone, 2-methoxyestradiol, and synthetic oligonucleotides inhibit its expression, acriflavine inhibits HIF dimerization, echinomycin inhibits its DNA binding activity and chetomin blocks its transcriptional activity (77). Alternatively, one could use hypoxia prodrugs (e.g., evofosfamide, tirapazamine**)** which are activated in hypoxic environments. Novel therapeutic strategies that target HIF-1 α and other key regulators will limit the capacity for the metabolic rewiring of breast CSCs and render these tumor-initiating cells more vulnerable to the varying conditions of the tumor microenvironment.

## Author Contributions

HW, BC, and JB drafted the manuscript, figure, and table. PM edited and conceived the manuscript figure and table.

### Conflict of Interest Statement

The authors declare that the research was conducted in the absence of any commercial or financial relationships that could be construed as a potential conflict of interest.
